# Application of Phylodynamic Tools to Inform the Public Health Response to COVID-19: Qualitative Analysis of Expert Opinions

**DOI:** 10.2196/39409

**Published:** 2023-04-21

**Authors:** Shannan N Rich, Veronica Richards, Carla Mavian, Brittany Rife Magalis, Nathan Grubaugh, Sonja A Rasmussen, Simon Dellicour, Bram Vrancken, Christine Carrington, Rebecca Fisk-Hoffman, Demi Danso-Odei, Daniel Chacreton, Jerne Shapiro, Marie Nancy Seraphin, Crystal Hepp, Allison Black, Ann Dennis, Nídia Sequeira Trovão, Anne-Mieke Vandamme, Angela Rasmussen, Michael Lauzardo, Natalie Dean, Marco Salemi, Mattia Prosperi

**Affiliations:** 1 Department of Epidemiology College of Public Health and Health Professions University of Florida Gainesville, FL United States; 2 Department of Epidemiology College of Medicine University of Florida Gainesville, FL United States; 3 Department of Pathology, Immunology, and Laboratory Medicine College of Medicine University of Florida Gainesville, FL United States; 4 Department of Epidemiology of Microbial Diseases Yale School of Public Health New Haven, CT United States; 5 Department of Pediatrics College of Medicine University of Florida Gainesville, FL United States; 6 Spatial Epidemiology Lab Université Libre de Bruxelles Bruxelles Belgium; 7 Department of Microbiology, Immunology and Transplantation Rega Institute KU Leuven Bruxelles Belgium; 8 Department of Preclinical Sciences University of the West Indies St Augustine Trinidad and Tobago; 9 Florida Department of Health in Alachua County Gainesville, FL United States; 10 Division of Disease Control and Health Protection Florida Department of Health Tallahassee, FL United States; 11 Division of Infectious Diseases and Global Medicine Department of Medicine College of Medicine, University of Florida Gainesville, FL United States; 12 Pathogen and Microbiome Institute Northern Arizona University Flagstaff, AZ United States; 13 School of Informatics, Computing, and Cyber Systems College of Engineering, Informatics, and Applied Sciences Northern Arizona University Flagstaff, AZ United States; 14 Pathogen and Microbiome Division Translational Genomics Research Institute Flagstaff, AZ United States; 15 Chan Zuckerberg Initiative Redwood City, CA United States; 16 Division of Infectious Diseases University of North Carolina at Chapel Hill Chapel Hill, NC United States; 17 Division of International Epidemiology and Population Studies Fogarty International Center National Institutes of Health Bethesda, MD United States; 18 Department of Microbiology, Immunology and Transplantation Rega Institute for Medical Research, Clinical and Epidemiological Virology KU Leuven Leuven Belgium; 19 Center for Global Health and Tropical Medicine Instituto de Higiene e Medicina Tropical Universidade Nova de Lisboa Lisbon Portugal; 20 Vaccine and Infectious Disease Organization University of Saskatchewan Saskatoon, SK Canada; 21 Department of Biostatistics College of Public Health and Health Professions University of Florida Gainesville, FL United States; 22 Department of Biostatistics College of Medicine University of Florida Gainesville, FL United States

**Keywords:** COVID-19, SARS-CoV-2, molecular epidemiology, genomic surveillance, variants, pandemic, phylogenetic, genomic, epidemiology, data, virology, bioinformatics, response, phylodynamic, monitoring, surveillance, transmission

## Abstract

**Background:**

In the wake of the SARS-CoV-2 pandemic, scientists have scrambled to collect and analyze SARS-CoV-2 genomic data to inform public health responses to COVID-19 in real time. Open source phylogenetic and data visualization platforms for monitoring SARS-CoV-2 genomic epidemiology have rapidly gained popularity for their ability to illuminate spatial-temporal transmission patterns worldwide. However, the utility of such tools to inform public health decision-making for COVID-19 in real time remains to be explored.

**Objective:**

The aim of this study is to convene experts in public health, infectious diseases, virology, and bioinformatics—many of whom were actively engaged in the COVID-19 response—to discuss and report on the application of phylodynamic tools to inform pandemic responses.

**Methods:**

In total, 4 focus groups (FGs) occurred between June 2020 and June 2021, covering both the pre- and postvariant strain emergence and vaccination eras of the ongoing COVID-19 crisis. Participants included national and international academic and government researchers, clinicians, public health practitioners, and other stakeholders recruited through purposive and convenience sampling by the study team. Open-ended questions were developed to prompt discussion. FGs I and II concentrated on phylodynamics for the public health practitioner, while FGs III and IV discussed the methodological nuances of phylodynamic inference. Two FGs per topic area to increase data saturation. An iterative, thematic qualitative framework was used for data analysis.

**Results:**

We invited 41 experts to the FGs, and 23 (56%) agreed to participate. Across all the FG sessions, 15 (65%) of the participants were female, 17 (74%) were White, and 5 (22%) were Black. Participants were described as molecular epidemiologists (MEs; n=9, 39%), clinician-researchers (n=3, 13%), infectious disease experts (IDs; n=4, 17%), and public health professionals at the local (PHs; n=4, 17%), state (n=2, 9%), and federal (n=1, 4%) levels. They represented multiple countries in Europe, the United States, and the Caribbean. Nine major themes arose from the discussions: (1) translational/implementation science, (2) precision public health, (3) fundamental unknowns, (4) proper scientific communication, (5) methods of epidemiological investigation, (6) sampling bias, (7) interoperability standards, (8) academic/public health partnerships, and (9) resources. Collectively, participants felt that successful uptake of phylodynamic tools to inform the public health response relies on the strength of academic and public health partnerships. They called for interoperability standards in sequence data sharing, urged careful reporting to prevent misinterpretations, imagined that public health responses could be tailored to specific variants, and cited resource issues that would need to be addressed by policy makers in future outbreaks.

**Conclusions:**

This study is the first to detail the viewpoints of public health practitioners and molecular epidemiology experts on the use of viral genomic data to inform the response to the COVID-19 pandemic. The data gathered during this study provide important information from experts to help streamline the functionality and use of phylodynamic tools for pandemic responses.

## Introduction

SARS-CoV-2, the cause of COVID-19, has rapidly spread worldwide since its emergence in Wuhan, China, in December 2019 [1]. As of January 2022, the virus had contributed to more than 366 million infections and 5.6 million deaths worldwide [2]. The first genomic sequence of SARS-CoV-2 was published in record time in January 2020, formally establishing COVID-19 as a novel disease [3]. In the intervening 2 years, over 7 million SARS-CoV-2 genome sequences have been deposited in the Global Initiative on Sharing All Influenza Data (GISAID) repository [4], which has served as the primary open access sequence archive for sharing SARS-CoV-2 sequence data since the start of the COVID-19 pandemic. The collection of SARS-CoV-2 viral genomes enables genomic surveillance of genetic variation over time and the discovery of new and consequential mutations in key regions of the virus’ genome.

Genomic surveillance of emergent pathogens, such as SARS-CoV-2, informs our understanding of their origins [5,6], transmission dynamics [7], spatial spread [8], and the emergence of variants [9], particularly when viral genome data can be coupled to standard surveillance data [10]. Given the widespread transmission of SARS-CoV-2 and the severity of COVID-19, researchers worldwide have worked swiftly to investigate SARS-CoV-2 sequence evolution and spread through molecular epidemiology methods [11]. Bioinformatics and data visualization tools that use phylogenetic trees, such as Nextstrain [12], COVID-19 CoV Genetics (COVID-19 CG) [13], and Ultrafast Sample placement on Existing tRees (UShER) [14], have rapidly gained popularity. Since the beginning of the pandemic, the Nextstrain tool has traced SARS-CoV-2 by adding sequences in real time to a global epidemic tree, aiding with the localization of new infections within existing clusters. For example, heavily subsampled, custom Nextstrain analyses have been used to investigate the possible patient 0 in Italy [15] and undetected transmission at the beginning of the pandemic in the United States [16]. Aside from COVID-19, the platform has also been used to forecast influenza A strains for vaccine predictions [17] and to create situation reports for the Ebola outbreak in the Democratic Republic of the Congo in 2018 [18]. However, these tools are less equipped to forecast the growth of viral clusters and their reliability is heavily affected by sampling bias [19-22]. Even Bayesian phylogeography, which integrates spatial and epidemiological data and is more computationally expensive than the tree-building algorithms used in online tools, only provides a reconstruction of past dynamics [23].

Although the existing mainstream phylogenetic tools are useful to provide insights into the molecular epidemiology of SARS-CoV-2, their utility in informing the ongoing response to COVID-19 among public health practitioners on the ground needs to be explored. Additionally, consumer-level input regarding what features are desired is unknown. Qualitative research methods are a useful approach to explore the perceptions and opinions of complex topics and engage stakeholder buy-in and are increasingly being performed in public health research [24,25]. Focus groups (FGs) are 1 type of qualitative method in which participants, who are homogeneous with respect to a shared area of expertise or experience, are guided through a structured discussion by a trained moderator [25]. The group dynamics elicited by this strategy can serve as a proxy informant for the community [25].

The objective of this study was to convene experts in public health, infectious diseases, virology, bioinformatics, and molecular epidemiology—many of whom were actively working on the COVID-19 response at the time of their participation—into FGs to discuss and report on the application of phylodynamic tools to inform the ongoing public health response to COVID-19. In total, 4 FGs occurred between June 2020 and June 2021, covering both the pre- and postvariant strain emergence and vaccination eras of the ongoing COVID-19 crisis.

## Methods

### Study Population

Participants included national and international academic and government researchers, clinicians, public health practitioners, and other stakeholders recruited through purposive and convenience sampling by the study team and expanded through snowballing (ie, in which invited participants could suggest others). The study team contacted professionals in the field whose contact information (eg, email address) is publicly available on their respective institutions' websites.

### Ethical Considerations

The study was approved by the University of Florida Institutional Review Board (reference number: IRB202000840). The FG participants provided written informed consent prior to participation. No compensation was provided.

### Study Instrument

Open-ended questions were developed to prompt discussion (Table 1). Questions were created to guide the discussion of specific topics and to gather diverse insight from a range of experts. The moderator was free to probe with additional questions to seek clarity or depth in participants’ responses [25]. FGs I and II concentrated on phylodynamics for the public health practitioner, while FGs III and IV discussed the methodological nuances of phylodynamic inference. We conducted 2 FGs per topic area to increase data saturation (ie, the point in data collection at which no new insights are added to the discussion [24]). Ten days before each FG, the facilitators circulated the study materials (eg, agenda, papers) and prearranged questions.

**Table 1:** Open-ended questions for the FG^a^ discussions.

FGs	Questions
FGs I and II: phylodynamics for the public health practitioner (June and October 2020)	What is your perception of the utility of phylogenetics to investigate epidemics of infectious respiratory illnesses, such as COVID-19? What are some hypothetical scenarios in which phylogenetic analysis can aid public health departments in preventing and controlling the spread of SARS-CoV-2? Tell us about your use (or knowledge of) phylogenetic tools for public health (eg, Microbe-Trace, HIV-TRACE, or Nextstrain). What do you like and dislike about them? What additional infrastructure is needed to enable SARS-CoV-2 phylogenetic analysis by health departments? What type of epidemiological data do you envision should be paired with sequence data to enable public health investigations of SARS-CoV-2 spread?
FGs III and IV: phylodynamic inference (May and June 2021)	Tell us about your use (or knowledge of) phylogenetic tools for public health (eg, Microbe-Trace, HIV-TRACE, or Nextstrain). What do you like and dislike about them? Tell us how you identify clusters/clades of interest in your analyses. Are you more inclined to assess trends across all identified clusters or focus on individual clusters? Why is that? What are the criteria you use to prioritize clusters? To what extent, if any, does heterogeneous sampling (i.e., sampling bias) impact your conclusions based on these results? How do you currently account for it? How would you envision a software program capable of overcoming it without downsampling/losing information?

^a^FG: focus group.

### Study Procedures

The FGs were conducted remotely via University of Florida’s Zoom [26]. There was a primary moderator whose role was to pose the questions, facilitate discussion, and ensure each participant had an equal chance to participate. A secondary moderator took notes, audio recorded each session, and monitored the chat window for written responses. Participants were encouraged not to include identifying information during the discussions. Audio recordings were transcribed verbatim by the professional transcription service Rev [27]. Transcripts were screened for accuracy by a member of the research team, and all identifying information was removed before analysis.

### Data Analysis

Data analysis occurred iteratively using a thematic qualitative inductive content analysis process [28]. This approach was selected to allow novel themes to emerge from the data independent from any preconceived categories. The knowledge generated was based on the FG participants’ unique viewpoints grounded in the qualitative data. Two reviewers from the research team read through the FG transcripts separately to identify provisional themes and then convened to discuss the findings and arrive at definitions for the agreed-upon themes. The researchers then separately coded all FG transcripts according to the identified themes, after which they met to discuss and resolve any discrepancies. Coding was accomplished using NVivo (QSR International) [29].

## Results

### Participant Details

Of the 41 individuals invited, 23 (56%) agreed to participate. There was an average of 5-6 participants (mean 5.75, SD 2.3) in each FG. The discussions lasted on average 52.6 (SD 10.1) minutes. Across the 4 FG sessions, 15 (65%) of the participants were female, 17 (74%) were White, and 5 (22%) were Black. Participants were described as molecular epidemiologists (MEs; n=9, 39%), clinician-researchers (n=3, 13%), infectious disease experts (IDs; n=4, 17%), and public health professionals (PHs) at the local (n=4, 17%), state (n=2, 9%), and federal (n=1, 4%) levels. They represented multiple countries in Europe, the United States, and the Caribbean. Nine major themes arose from the discussions (Table 2).

**Table 2:** Operationalized themes and definitions.

Theme	Definition
Translational/implementation science	Application of phylodynamic research findings into policy and public health practice
Precision public health	Targeting interventions toward specific populations (or “clusters”)
Fundamental unknowns	Lack of knowledge due to the nature of an emerging infectious disease
Proper scientific communication	Rapid dissemination and proper interpretation of SARS-CoV-2 molecular data and phylodynamic analyses
Methods of epidemiological investigation	Traditional tracing (case investigation/contact tracing) versus molecular/phylogenetic tracing, and methodological nuances of phylogenetic/phylodynamic studies
Sampling bias	Bias in the way the samples were collected, or sequence data were shared, and resulting implications
Interoperability standards	Having consistent rules for storing, publishing, and sharing sequence data between stakeholders and researchers
Academic/public health partnerships	Building relationships and assigning complementary/noncompeting roles between academic and public health partners
Resources	Funding, equipment, and ability and availability of personnel to conduct molecular epidemiology investigations

#### Translational and Implementation Science

The application of phylodynamic research findings into policy and public health practice, labeled as “translational and implementation science” in the qualitative analysis, emerged as a common theme from the FG discussions. Many of the participants felt that phylogenetic data are important for evaluating public health policies:

It's important when it comes to evaluating policy, to see what policies may have been effective. Do border closures really stop transmission from one place to another? You could look at policies like that with this type of data.

FG2, PH local

Additionally, participants remarked (particularly when thinking about border closures or travel-related cases) that having a better understanding of the virus’s movement across state and county lines would facilitate better communication about interstate disease transmission to the public. Moreover, using phylogenetics to refine the identification of where transmission is occurring can inform the creation of place- or setting-specific policies designed to mitigate transmission:

This would be really helpful for identifying where transmission is occurring and then figuring out what it is about those facilities or places that is facilitating transmission so we can inform policy that tamps down that transmission.

FG2, PH local

In contrast, a minority of the participants felt that the relative impact of phylodynamic analyses to inform an ongoing public health response was low due to precedent:

In terms of utility and the practical application, I would say my perception is low, because, at the local health department level, we don't utilize the existing phylogenetic information we have for other pathogens. So why would COVID be terribly different?

FG1, clinician-researcher

Other reflections considered the clinical utility of phylogenetics in the face of other clinical concerns that also require funding and resources:

How is this going to help me prevent cases, treat cases, find cases, anticipate cases, and actually do an intervention where I can show that I’m using my clinical resources to stop something worse from happening?

FG1, clinician-researcher

#### Precision Public Health

Another theme to emerge was the notion of tailoring public health interventions toward specific populations (or “clusters”), which we termed “precision public health.” The participants remarked that having the phylogenetic information allows one to evaluate the role that any 1 outbreak might play in the larger spread, which can inform mitigation strategies. Participants imagined a reality where interventions could be tailored to the dominant strains circulating in a community:

I would love to know about the virulence, transmissibility, and pathogenicity of each strain, because that will change what I do practice-wise, as long as it can be in real-time. So, quick PCR says: “Presence of virus?” Yes. “Strain?” X. Activity falls into “this” category. That would be heaven in terms of control.

FG1, clinician-researcher

Other reflections considered the value of using phylogenetics to resolve transmission events and distinguish between probable transmission settings to aid with setting-specific accountability and prevention.

If you have 6 cases, let's say, at a meatpacking plant, and then you have family members in the household also testing positive, is the focus of transmission actually the work setting, or are people becoming infected by their household members who are also going to school or working in frontline industries. I think that's going to be important, as employers try to be accountable or dodge that accountability.

FG2, ME researcher

Participants additionally discussed variant tracking to understand the proportions of certain variants circulating in the community at any given time:

Using phylogenetics to follow the trend of variants of interest and variants of concern to see which proportion of infection at a particular time [is] a particular variant and how variants are competing with each other and taking over.

FG3, ME researcher

The participants also considered how phylodynamic tools can aid with investigating pockets of outbreaks as the disease transitions from an epidemic to an endemic state:

If it was to establish itself as an endemic disease, then phylogenetics will be very important in aiding public health departments to investigate pockets of outbreaks. It can answer whether transmission occurred some time ago or if it was imported from another region. That will be very important to investigate.

FG1, ID researcher

#### Fundamental Unknowns

Many of the participants discussed the problem of fundamental unknowns or the lack of knowledge due to the nature of an emerging infectious disease and how this may impact transmission chain analyses and variant tracking:

I think now in the acute phase of the pandemic, we don't know enough about how the virus evolves and how much genetic diversity is accumulated between transmission events to really use a [phylodynamic] tool to trace transmission from person to person.

FG1, ID researcher

I think that you can try to start looking for variants that might be behaving differently, but we also really need to be cautious about that. It can seem as though a particular variant is infecting a certain group of people, but what's really hard is to try to figure out whether that's something inherently functionally different about the virus or whether it's something that's being driven by founder effects or behavior or something like that.

FG2, ID researcher

In addition to citing conceptual concerns about the technologies’ capabilities, participants also expressed difficulty in convincing decision makers to act now to prevent an outcome that is not yet certain:

It's really difficult to get decision makers to properly understand what's coming in the next months. They think, why do they have to make decisions now for something that is only going to become clear after a few months?

FG3, ME researcher

Other participants felt that the collection and analysis of genomic sequence data will be invaluable for answering many fundamental unknown questions. Participants discussed how genomic analyses can provide insight into the immunologically important epitopes of the SARS-CoV-2 spike protein, including the receptor-binding domain, and other critical motifs for neutralization to help preserve the efficacy of currently available vaccines.

#### Proper Scientific Communication

Another theme to emerge across most of the discussions was related to proper scientific communication. Many of the participants urged the importance of disseminating proper interpretations of SARS-CoV-2 molecular data and phylodynamic analyses:

We need to be careful to not make grandiose conclusions about why an outbreak happens or give too much weight to it. It's one of many types of data that can be blown out of proportion or interpreted incorrectly.

FG2, ID researcher

This idea of toning down conclusions and putting findings into perspective to prevent incorrect interpretations was echoed by many participants. One participant further expanded to consider the implications of how easily accessible some phylodynamic tools have become, giving people access to more phylogenetic data than ever before:

It's not just the tool that is important; it's also the people using the tool and how they make sense of the tool in a public forum. Journalists see all these nice graphs from Nextstrain, and they make their own conclusions, but they are not epidemiologists. They just want a headline for their newspaper. These are all becoming very democratic tools, and everyone has access to them, but you need to put things in context and warn for misinterpretations of the data.

FG2, ME researcher

#### Methods of Epidemiological Investigation

Another theme that emerged was related to participants’ experiences with traditional epidemiological investigations, that is, tracking transmission through case investigations and contact tracing to molecular approaches to investigation, as well as the methodological nuances of phylogenetic/phylodynamic studies:

We have a lot of people who don't want to be forthcoming. They feel like they're protecting their friends. They don't want to talk about where they've been, different parties they've been to, because of certain policies and just social desirability bias. So, having a more objective tool for evaluating transmission would be really powerful. There is, I think, a lot of fear of retaliation, more perceived than real, but we do need to collect this information, and this is another way to go about it without having to rely on them to be entirely forthcoming.

FG2, PH local

Another researcher added that phylogenetic data can help refine transmission events:

I think it is really important when you have something that is broadly transmitting throughout the community, but which there may actually be different associated factors that are important for transmission, whether that is community transmission in schools, just baseline transmission, [or] workplace transmission. So I think that the phylogenetic data [are] helpful for really narrowing in areas where you have a lot of ongoing transmission, relating cases to those clusters when it's not necessarily clear from epidemiologic interview data, and then also potentially, when you have those more sensitively defined clusters, you can improve your estimates of important epidemiologic parameters, like R0, that could be more biased if you are including cases as part of a cluster definition that really don't belong in that cluster.

FD2, ME researcher

Others argued that contact tracing data should still be considered the ground truth, although not without caveats:

The shoe-leather epi is critically important for identifying clusters of cases. We try not to let the genomics inform that and rather let the epidemiology and contact tracing inform it. Then, we just use the genomic similarity to help to confirm what the epi team has already put together or to show that maybe something is not the case. It's pretty clear when things are different lineages that there's not a direct transmission between them. But when they're identical genomes and you have epidemiological information that would suggest that one person may have transmitted to somebody else, then at least our data helps to confirm that hypothesis, but it doesn't prove anything. It's, I think, a really complex space where you have 2 separate fields that are trying to figure out how to work together and trying to understand the uncertainty in both aspects of it. For example, I treat contact tracing as [the] ground truth when doing this work, but there are a lot of biases within contact tracing too that maybe I don't quite understand. I think maybe some frameworks to help to better combine the 2 aspects would be really good for outbreak investigation.

FG3, ME researcher

#### Sampling Bias

Sampling bias, or bias in the way samples were collected or sequence data were shared, and the resulting implications of this, was another emergent theme. Most of the participants acknowledged sampling bias as 1 of the major causes of data misinterpretation in phylodynamic analyses:

I would say that the sampling bias is a huge issue in phylodynamic, phylogeographic, and molecular epidemiology in general. It has to be acknowledged. It has to be treated. It has to be investigated before and after the analysis.

FG4, ME researcher

When you do phylogenetic analyses or phylogeography, you focus on the evolutionary relationships or the dispersion history of the lineages that you sampled. If you then want to generalize what you found on the entire epidemic, you need to be confident about the representativeness of your sample compared to the epidemic.

FG4, ME researcher

Some of the participants reflected on lessons learned from the Ebola pandemic:

I think it (genomic data) can lead us astray if it's not really analyzed in the proper context. I'm thinking of the Ebola virus and the 2014 epidemic that occurred in West Africa. One hypothesis was that the virus in West Africa had a higher mutation rate than other Zaire ebolaviruses, which turned out not to be true. It was a very hot topic of discussion, that somehow this higher mutation rate could explain why Ebola had emerged in a part of Africa that was very geographically distant from any place it had ever been observed before. In reality, it's probably just that we weren't surveilling ebolavirus in wildlife well enough to understand that it was already in West Africa, in a different population, probably in bats, then in central Africa.

FG2, ID researcher

So, the higher number of mutations that were observed early on in the Sierra Leonean portion of that outbreak is not untrue, but I think it's not like the biology of the virus is different. It's really about sampling, the sampling frame, and the fact that you're sampling variants that would be selected out likely over broader timescales. I think that that brings up a thing that is true really across pathogens that you do this analysis on, and that I think we're seeing with COVID-19 as well, which is all the pieces on the board move, and the rate at which you've sampled really matters.

FG2, ME researcher

Other examples of sampling bias that were brought up were related to missing high-risk clusters due to testing avoidance and only catching those infections in individuals who follow public health guidelines (eg, required mitigation testing):

We see that there is a high overlap among people who have a high vaccine hesitancy, people who disregard masks, distancing, small social network behaviors, and people who avoid testing when they are required to. I am concerned that the samples that we're getting are going to be limited to those who either have symptoms or are following all public health guidelines. I think there's a real risk for undersampling the highest risk clusters.

FG3, ID researcher

Temporal bias was another issue brought up:

In an ideal world, you would have a sampling that matches the spatial-temporal intensity of the epidemic. So, if you have more cases during a specific time period, you should also have more samples from that time period. The bias you have and the potential impact of the bias you have [depend] on the research questions. Some biases are not that important. Others are, depending on the question you have.

FG4, ME researcher

The participants discussed issues related to big data and the need to downsample (ie, removing sequences) to subset or condense their background/reference data sets to run many types of phylodynamic analyses. They imagined having a tool that allows one to tailor the sample selection process would be useful:

In the era of SARS-CoV-2, we have so much data that it's impossible to use most of these programs without downsampling. We are getting to the point where we even have to downsample our own data. Sometimes, that's okay, and sometimes, it can cause issues. Which brings in the question of, how do you downsample properly?

FG3, ME researcher

I think 1 of the things that could be improved in Nextstrain is the background data set because unless you have a specific instance for your region, the conclusions you'll be making from the general background data set available will be quite biased and probably wrong. If the platform could automatically change the background data set according to a lineage that you're looking for or something like that, that would be very useful.

FG3, ME researcher

The reason for sequencing can also impact findings, as remarked by 1 participant:

One thing that is an issue when you're using data generated by other groups is what is the reason for sequencing? That can significantly impact your findings. For example, if a group is only doing sequencing for an outbreak investigation, you're going to have clusters of related genomes, which is going to make those look like they're higher frequency than if you're just doing a random subset. Even a random subset of samples is not really random, because they have to meet certain criteria for sequencing anyway and often sequencing is based on convenience, not necessarily representativeness.

FG3, ME researcher

And then on top of that, you have targeted sequencing of S-gene target failures that happened for a while, which then increase the proportion of B.1.1.7 relative. There's a lot of things that go into this that make it sometimes hard just to correlate a large collection of data to infer trends.

FG3, ME researcher

Incorporating epidemiological and contact-tracing data was 1 method discussed to help ameliorate sampling biases:

I would love to have some good way of trying to control for ascertainment bias. I think if there was really a nice way of incorporating contact tracing and other kinds of epidemiological data into the sequence data, I think that would have a tremendous impact, and it's something we worry about all the time but don't have a great solution to.

FG3, ME researcher

The participants discussed other ways to address sampling bias in the discussions, including to homogenize the data set before the analysis, to make sure it is adequately representative of the epidemic and through post hoc approaches to assess to what extent sampling bias had an impact on the outcome of the analysis:

Let's say that you have heterogeneous sampling and, before starting the analysis, you subsample your data sets according to local incidence. Then, you want to have a number of sequences per locality that is proportional to the relative importance of the epidemic at that location. So, 1 way to deal with that is to relate local incidence with the number of sequences that you subsample by location. Or try to homogenize your data set prior to the analysis. So, you have to obtain a subsampling that is related to the relative importance of the incidence in true space and time.

FG4, ME researcher

#### Interoperability Standards

When considering many of the obstacles for storing, publishing, and sharing sequence data between stakeholders and researchers, the FG participants discussed the desire for having consistent rules and even a centralized system. This theme was coded as “interoperability standards.” Participants believed that the lack of uniform and routine collection of SARS-CoV-2 genomic sequences is a missed opportunity. They argued that a centralized system for specimen collection and reporting of sequences could help avoid duplicity in data entry and harmonize phylogenetic data with clinical, epidemiological, and demographic data. They also reasoned that this could improve relations between the different levels of public health:

I think there's a desire to build a system at the federal level that the states can access, improving that interconnectivity both between local health departments and states, and states into the federal level.

FG1, PH state

As discussed within the theme of sampling bias, when choosing reference sequences from public data repositories, knowing the reason for sequencing is critical but rarely known. Interoperability standards for sequence data submission could help ameliorate this:

Again, this is really going to have to be at the point of GISAID or GenBank or any other place that has a repository, but with set definitions, there could be a metadata field that has a dropdown menu perhaps that allows you to say “outbreak investigation,” “vaccine breakthrough,” etc. Whenever you are targeting samples for sequencing, aside from just general surveillance, you could indicate the justification for outside groups who may be interested in pulling your [sequence] data for use in their own analyses.

FG3, ME researcher

Some of the participants imagined where this system could be housed:

Maybe it's housed at the NIH or somewhere else within [the] DHHS and where the epi data and the genomic data are all housed. And that when a researcher is trying to go in and get a data set, you could have collision prevention. You only want at most 2 sequences from 1 outbreak scenario or 5 sequences within a week from a particular college campus so that the researcher isn't or the public health person isn't actually looking at the very specific metadata that's housed, but can prevent this overrepresenting one group over another, if that's the goal.

FG3, ME researcher

The centralized system could generate custom reports and data extracts based on filtering criteria selected by the user. It could also host genomic assembly information:

It's important to tie genomic assembly information to the genomic data as well, in terms of what types of tools were used to assemble the genome, whether it's a minor variant or a consensus level call or all of those things, because those are actually really important parts of the analysis. When that gets lost, it really impacts the usability of the data more generally.

FG2, ME researcher

Although a centralized system could solve many of the issues identified, some of the participants added that building such a system would require a lot of organizational work and cooperation across different groups. Additionally, legislation, regulatory frameworks, or a multitude of contractual agreements would need to be put in place to facilitate data sharing and communication with public health authorities across states and territories.

#### Academic and Public Health Partnerships

Academic and public health partnerships were another theme to emerge, which we defined as building relationships and assigning complementary/noncompeting roles between academic and public health partners. Many of the participants were actively involved with generating regular reports for the local health departments, tribal nations, universities, and other external partners and emphasized the importance of forming strong relationships with these entities:

We can think of infrastructure, not in terms of who's doing what where, but the relationships between academia and the health department.

FG1, ID researcher

When discussing who should be responsible for conducting routine molecular surveillance, many of the participants felt that it depends on who has the skillset. They thought that it is easier to recruit the type of talent that you need to universities rather than to health departments:

I think it's unrealistic to expect the health departments to have and maintain that level of [phylogenetic] expertise. You're going to have regional versus county versus state issues. And maintaining that capacity is going to be difficult. And then also, this will be a rapidly evolving field, and it'll be a lot easier to recruit the type of talent that you need to universities, rather than to health departments. And then also, you get the infrastructure that health departments desperately need, and that infrastructure is better relationships with the academic centers.

FG1, clinician-researcher

Others cited issues with the adoption of existing phylodynamic tools within the public health sector due to the limited infrastructure for computational power. Overall, the participants felt that having strong public health–academic partnerships is essential to accomplish this type of work. The applied public health sector should be identifying the questions that need answers, while the academics should focus on the methodological nuances of the analyses and the bigger picture:

I think in terms of the academic and public health joint participation in these issues, they have to come together to focus on the public health questions of importance. And I say sometimes academics have an important contribution to make to that discussion because sometimes people so involved in their fieldwork don't think about the other things that could be important.

FG1, ID researcher

The consensus of the groups was that contracting the work out to academics may be the best approach to maintaining expertise and staying on the “bleeding edge of the technology” (FG1, PH federal).

#### Resources

A recurring theme related to funding needs, equipment requirements, and the ability and availability of personnel to conduct molecular epidemiology investigations—collectively termed “resources”—was debated throughout many of the FG discussions. Some participants doubted the need for phylogenetics when performing mitigation testing. Others considered the limitations of the current infrastructure to run these types of analyses at public health departments:

I think that in terms of the infrastructure at the health departments, obviously personnel and expertise are needed. Epidemiologists are pretty few and far between right now, and so having persons who understand these data and how they can be used is paramount, because it's no point in having all of these data and these analyses if we don't understand how to use them. Secondly, I would say that it depends on whether or not the expectation for the sampling is to be on the public health system or if it is on the health care system is something else too, and maybe increasing the capacity at the public health labs to do these analyses is also needed.

FG2, PH state

I have to say that because of constraints with manpower, very few of us working on this, we really have been restricted to just looking for lineage assignments and seeing whether we have variants of concern, mutations of concern. All the other types of analyses that we would really love to do have not been possible yet, but we'll get there.

FG3, ME researcher

One participant commented on the lack of resources available to tap into existing data:

There's lots of data, reams of data, some of it from just straight up shoe-leather epidemiology, and we do nothing with it at the local level because the utility is not really clear, and there are not the resources to be able to tap into it.

FG1, clinician-researcher

Financial costs and timeliness were other key resource issues identified:

I think that one piece that was brought up that's really important is the timeliness and availability of the data for us to be able to apply it in an outbreak situation. I think that's going to be key. I'm not sure if there's lab capacity to be able to do that.

FG1, PH local

I think it depends on what the turnaround time would be between when the samples are collected, between when we get reports back, and how granular the data would get.

FG2, PH local

The collection of these data to actually come up with these phylogenetic clusters is, I feel, a very difficult thing to do. Who's going to pay for those tests, and do we have the capacity to actually obtain those specimens and run those in labs at the volume of testing that we've been doing in the state?

FG2, PH state

## Discussion

### Principal Findings

Amid the global public health crisis presented by COVID-19, researchers and scientists have strived to collect and analyze genomic data to inform public health decision-making in real time. Open source phylogenetic and data visualization platforms for monitoring SARS-CoV-2 genomic epidemiology, such as Nextstrain [12], have rapidly gained popularity for their ability to illuminate spatial-temporal transmission patterns worldwide. However, the utility of such tools to inform public health decision-making for COVID-19 in real time remains to be explored. In this study, we detailed the perspectives of experts in both academic and public health settings regarding the utility of phylodynamic tools for the public health response to COVID-19. Discussions were hosted across the pre- and postvariant and vaccination eras of the crisis. The overall participation rate was 56%, which is comparable to previous FG studies that recruited stakeholders and professionals across health disciplines [30,31]. The diverse group of participants represented a wide variety of expertise on the topic, including experts involved in the COVID-19 response at the time of their participation.

A variety of themes emerged during the FG discussions. Participants were optimistic about the ability of phylodynamic tools to track the spatial spread of the virus and to resolve the transmission patterns. Using these types of data to evaluate policy, such as the impact of border closures on transmission, was an important feature cited by many participants. A prior phylogeographic analysis of the origin and spread of SARS-CoV-2 in Europe revealed that the virus had already spread to several European countries (ie, France, Germany, and Italy) prior to border closures [32], while another analysis conducted using data from Russia revealed that early border closures helped delay virus introductions from China [33].

The translation of phylodynamic analyses into public health action was another theme discussed at length. There were some differences in the responses of public health practitioners by level of public health. County public health practitioners were generally enthusiastic about the use of sequence data to aid in their investigations of local outbreaks. In contrast, state public health officials, while acknowledging the potential utility of phylogenetic studies, were concerned about the resources needed to conduct the analyses and which entity (eg, academic institutions vs public health departments) should be responsible. The participants emphasized the need for strong academic and public health partnerships to enable the highest-level science available at academic centers to conduct analyses requested by stakeholders. Participants also mentioned the key types of data that would ideally be attached to the genomic sequences to permit more in-depth analyses. The majority of participants agreed that phylodynamics will remain critical to answer key fundamental questions about virus transmissibility and immune evasion. They also imagined that public health responses could be tailored to a specific variant and that phylodynamic tools could be used to monitor pockets of outbreaks as the disease transitions from an epidemic to an endemic state. There are a few instances of phylogenetic data being used to inform COVID-19 public health decisions in real time that are documented in the published literature. A study in Wales used phylogeographic methods to demonstrate the impact of travel restrictions on SARS-CoV-2 transmission, subsequently leading to their reinstatement [34]. In the United States, a molecular epidemiology study revealed the introduction of the highly transmissible B.1.1.529 (Omicron) variant into several states [35]. This led to a reduction in the recommended isolation period for infected individuals to blunt the societal impact of the virus [36].

The causes and effects of sampling bias were another theme thoroughly discussed. Nonrepresentative samples have been an ongoing issue for SARS-CoV-2 analyses as they can directly influence phylodynamic inference and lead to inaccurate conclusions about virus dispersion dynamics, as previously reported by our group [37,38]. Recent examples of emergent SARS-CoV-2 variants, such as the identification of the Alpha variant in the United Kingdom and the Omicron variant in South Africa, highlight key issues with sampling bias (and associated surveillance bias) as the first location of detection is often blamed for the origin despite reports of previous cryptic circulation in other countries [39]. These examples also emphasize the importance of proper scientific communication, another key theme to arise during the FG discussions. Proper scientific communication was emphasized by several of the participants who were disappointed by the media’s reporting of SARS-CoV-2 variants. We believe that the use of molecular epidemiology for public health decision-making, using a transdisciplinary approach that involves policy maker input, will be an important area for future training.

The desire for interoperability standards was a unique theme to emerge from the FGs. The participants discussed the need for standard operating procedures for sequence storage and sharing to reduce biases with background sequence data sets and improve many of the resource issues identified in the “resources” theme. Challenges with the storage and analysis of the enormous amount of SARS-CoV-2 genome sequence data available were a major topic of discussion among the FG participants, echoing similar calls for collective resolution by other groups [40]. There were some topics discussed only briefly or not brought up at all during the FGs. For instance, the security and privacy of traditional epidemiology data types (eg, clinical, demographic, and social contacts) versus pathogen genomic data was a topic of limited discussion. Additionally, there was no discussion of the burden placed on individuals during traditional contact tracing to construct transmission chains, which can be avoided with genomic epidemiology approaches.

In summary, the data gathered during this study provide important information from leading experts in phylogenetic inference, as well as public health practitioners, to help streamline the functionality and use of phylodynamic tools for pandemic responses. As stated by the participants, successful uptake of these tools will require strong academic and public health partnerships. Among their many recommendations was the development of interoperability standards in sequence data sharing to ensure consistency in reporting and to reduce oversampling of nonrandom persons. They also urged responsible reporting of results to prevent misinterpretation by the media and the public. In addition to these recommendations, the participants highlighted key resource issues, including timeliness and cost, that will need to be addressed by policy makers in future epidemics.

### Limitations

This study had some limitations. The sample was relatively small and is not representative of all key experts involved in the COVID-19 response. We had minimal participation of individuals from low-income countries. These limitations may explain the limited discussion of certain issues, such as privacy preservation and the individual burden of contact tracing, that we anticipated. Participants were, however, diverse in their expertise, having served in many different capacities throughout the pandemic. Further, the methods of participant recruitment used are prone to bias, which may limit the generalizability of the study, though these methods of recruitment are common and often necessary to recruit experts for qualitative FGs [41]. Discussion prompts were shared with participants 10 days prior to the FGs, which may have resulted in bias caused by outside consultation with peers; however, this is unlikely as the conversations were driven by group discussion. Although an interrater reliability score was not calculated, coding was conducted by 2 researchers using an iterative and systematic process that involved independently coding prior to comparison, minimizing subjectivity [42]. Codes were discussed until 100% agreement was reached.

### Conclusion

To the best of our knowledge, this is the first qualitative study to characterize the perspectives of key experts regarding the utility of phylodynamic tools for the public health response to COVID-19. The data gathered during this study provide important information to guide the development of phylodynamic tools for pandemic responses. This information is critical to both policy makers and developers as they consider how to handle existing and emerging SARS-CoV-2 variants during the ongoing crisis.

## Abbreviations

FG: focus group
GISAID: Global Initiative on Sharing All Influenza Data
ID: infectious disease expert
ME: molecular epidemiologist
PH: public health professional

## Data Availability

Much of the data generated and analyzed during the study are included in this published paper in quotations. The remaining data generated are not publicly available due to potential issues with reidentification but may be available from the corresponding author upon reasonable request.
